# Editorial: Fluid teams

**DOI:** 10.3389/fpsyg.2024.1427375

**Published:** 2024-06-25

**Authors:** Gregory Funke, Michael Tolston, Tripp Driskell, August Capiola, James Driskell

**Affiliations:** ^1^Air Force Research Laboratory, Dayton, OH, United States; ^2^Florida Maxima Corporation, Orlando, FL, United States

**Keywords:** fluid teams, teams, groups, team performance, teamwork, team dynamics

Modern work contexts have given rise to *fluid teams* that differ from traditional teams. Such teams have become prevalent in contexts such as healthcare (Bell et al.; Grossman et al.), innovation teams in industry (Linhardt and Salas), and the military (Capiola et al., 2020), aviation (Sand, 2020), and in novel interactive environments such as the metaverse (Jarvenpaa and Keating). Driskell et al. (2023) describe fluid teams as comprised of four core characteristics: (1) team members are rapidly assembled to address an immediate problem, (2) members are assembled based on domain expertise and typically have no prior history or experience working together, (3) the team must begin work on a task that is immediate, time-critical, and of short duration, and (4) at completion of the task, the team disbands with little likelihood of further interaction.

Fluid teams differ from traditional teams in several ways. First, because they are rapidly assembled, often from across disciplines, team members may lack familiarity with their teammates (though they may have substantial knowledge regarding the structure of the team, their role within it, etc.). Second, the immediate nature of the task may provide little time for team members to orient themselves to one another. Third, stemming from the short time frame of the team's interaction, these teams do not have the opportunity to develop characteristics such as cohesion or well-developed shared mental models. Finally, the team dissolves upon task completion with no anticipation of future interaction.

One broad, overarching theme across the articles in this Research Topic is that fluid teams can confer both advantages and disadvantages. On one hand, it allows disparate teams of experts to be assembled on short notice to address urgent and time-restricted tasks irrespective of location or organizational affiliations, and can lead to greater flexibility and faster innovation. On the other hand, these conditions pose unique challenges in terms of team assembly, operation, and factors supporting effective teamwork. The articles presented in the Research Topic extend our understanding of this rapidly growing team configuration.

In a critical healthcare context, Bell et al. examine the challenges related to fluid teamwork in the neonatal intensive care unit (NICU). They note that NICU teams are multidisciplinary and experience frequent changes in membership as staff and other members join and leave the team. They address three broad challenges that can hinder effective teamwork and patient care in this context: (1) incorporating patient families into the healthcare team, (2) managing the medical hierarchy, and (3) facilitating effective patient handoffs across teams. Finally, they offer practical recommendations from team science to address these challenges.

Driskell et al. review the team composition literature to address considerations for forming fluid teams. They note that fluid teams are unique in that they are rapidly assembled to execute critical, time-limited tasks and are composed of members who typically have no prior experience working together, and who disband upon task completion. Their analysis focuses on the individual-level attributes of team members that support effective performance in this unique team context, with implications for composing fluid teams.

Grossman et al. provide an overview of the dynamics of fluid teams and utilized critical incident techniques and thematic analysis to examine fluid teams within a healthcare simulation. They sampled students who participated in high-stakes simulations and medical faculty who oversaw them to elucidate key factors that facilitate fluid team effectiveness. Their analysis illuminates critical themes including ambiguity and inconsistencies regarding team member roles, effective leadership, coordination difficulties, trust, and other factors. Recommendations for practice based on these themes are provided.

Hughes et al. conducted an analysis of team resilience in primary care teams, drawing on large-scale data from Patient Aligned Care Teams during the COVID-19 pandemic. They examine the extent to which teams maintain performance under adverse conditions and how team performance may be helped or hindered by team member fluidity. Their results shed light on the relationships between team turnover, coordination and performance in teams experiencing adversity.

Linhardt and Salas focus on innovation teams that generate and implement creative and novel ideas. They note that fluid team membership can benefit innovation in that the flexibility and new combinations of team members may support novel and innovative solutions, yet fluidity may also disrupt necessary teamwork processes. A conceptual framework of fluid membership in innovation teams is proposed, emphasizing the effects of knowledge integration and team reflexivity on building team resilience.

Research has suggested that joint problem-solving orientation (JPS) is a factor that promotes performance in fluid teams. Kerrissey and Novikov test this proposition in a large survey-based field study of patient care personnel in a healthcare setting. Their results provide support for a moderated mediation model in which JPS enhances performance directly and through mutual value recognition (MVR) as a mediator; JPS was most strongly related to MVR when expertise variety was high. This suggests that team fluidity may inhibit familiarity with other team members, but a joint problem-solving orientation may help team members recognize the value of other's contributions and promote team performance.

Jarvenpaa and Keating present a stimulating and forward-thinking analysis of opportunities and challenges that the metaverse might pose for fluid teams in organizations. Conceptualizing the metaverse as comprising virtual and physical environments that are integrated but distinct, they examine familiarity from three perspectives: interpersonal interactions that take place in the metaverse, the self and identity in virtual contexts, and time. Additionally, they propose potential research avenues to advance current understanding.

Driskell et al. provide an overview of the critical research gaps and opportunities in understanding fluid team performance. They outline a set of key Research Topics to further our understanding of fluid team performance in the areas of selection and composition of fluid teams, work and task design, and team member training. Finally, methodological and measurement challenges in studying fluid teams are identified.

## Author contributions

GF: Writing – review & editing, Writing – original draft. MT: Writing – review & editing, Writing – original draft. TD: Writing – review & editing, Writing – original draft. AC: Writing – review & editing, Writing – original draft. JD: Writing – review & editing, Writing – original draft.

## References

[B1] Capiola A. Baxter H. C. Pfahler M. D. Calhoun C. S. Bobko P. (2020). Swift trust in ad hoc teams: A cognitive task analysis of intelligence operators in multi-domain command and control contexts. J. Cognit. Eng. Dec. Making 14, 218–241. 10.1177/1555343420943460

[B2] Driskell T. Funke G. Tolston M. Capiola A. Driskell J. E. (2023). Fluid and virtual teams. Small Group Res. 29:10464964231216367. 10.1177/10464964231216367

[B3] Sand M. B. A. (2020). Can you trust someone you have never met? *Swift trust and self-disclosure in temporary teams* (unpublished thesis). Tromsø: UiT Norges Arktiske Universitet.28940348

